# A Novel Robotic Controller Using Neural Engineering Framework-Based Spiking Neural Networks

**DOI:** 10.3390/s24020491

**Published:** 2024-01-12

**Authors:** Dailin Marrero, John Kern, Claudio Urrea

**Affiliations:** Electrical Engineering Department, Faculty of Engineering, University of Santiago of Chile (USACH), Av. Víctor Jara 3519, Estación Central, Santiago 9170124, Chile; dailin.marrero@usach.cl (D.M.); claudio.urrea@usach.cl (C.U.)

**Keywords:** spiking neural networks, robotic control, NEF, Nengo

## Abstract

This paper investigates spiking neural networks (SNN) for novel robotic controllers with the aim of improving accuracy in trajectory tracking. By emulating the operation of the human brain through the incorporation of temporal coding mechanisms, SNN offer greater adaptability and efficiency in information processing, providing significant advantages in the representation of temporal information in robotic arm control compared to conventional neural networks. Exploring specific implementations of SNN in robot control, this study analyzes neuron models and learning mechanisms inherent to SNN. Based on the principles of the Neural Engineering Framework (NEF), a novel spiking PID controller is designed and simulated for a 3-DoF robotic arm using Nengo and MATLAB R2022b. The controller demonstrated good accuracy and efficiency in following designated trajectories, showing minimal deviations, overshoots, or oscillations. A thorough quantitative assessment, utilizing performance metrics like root mean square error (RMSE) and the integral of the absolute value of the time-weighted error (ITAE), provides additional validation for the efficacy of the SNN-based controller. Competitive performance was observed, surpassing a fuzzy controller by 5% in terms of the ITAE index and a conventional PID controller by 6% in the ITAE index and 30% in RMSE performance. This work highlights the utility of NEF and SNN in developing effective robotic controllers, laying the groundwork for future research focused on SNN adaptability in dynamic environments and advanced robotic applications.

## 1. Introduction

The development of the smart factory paradigm, as proposed by Industry 4.0, demands technologies capable of adapting and evolving to meet the growing needs of the industry [1]. To achieve this, increasingly intelligent robotic systems are required, capable of learning new tasks with greater skill and autonomy in decision making. Such systems evolve into self-aware and self-adapting systems for new products and manufacturing processes [2].

One of the main objectives pursued in this context is enabling robots to perceive the environment and act in the real world almost as efficiently and autonomously as humans [3]. This action requires optimizing processing in such a way that robotic systems can integrate a large amount of information, take the corresponding control action in real time, and self-adapt to changing environmental conditions.

These needs are not always easy to fulfill with traditional control strategies, which employ numerical methods based on kinematics and dynamics equations. Typically, these methods are designed for a specific purpose or task and encounter difficulties adapting to scenarios with changing conditions.

On the other hand, one of the alternatives adopted is controllers based on conventional Artificial Neural Networks (ANN). In [4], the authors develop an adaptive neural network control method to achieve effective trajectory tracking for robotic arms. The use of neural networks helps handle uncertainties within the control system and external interference, resulting in improved overall performance, as validated by experiments.

The article presented in [5] presents a vision-based robot control method that integrates convolutional neural network (CNN) object detection, specifically focusing on robots with an eye-in-hand configuration. The approach employs real-time CNN detectors like Yolo to generate task variables based on bounding box parameters, addressing chattering with LSTM. The vision-based controller ensures stable object tracking within the camera’s field of view, as verified by experimental results.

A self-tuning PID controller based on ANN is proposed in [6] combining traditional PID control with artificial intelligence for robust performance. The neural network enables dynamic adjustments to PID controller parameters, particularly useful for systems with time delays and transportation lag. However, concerns about the vanishing gradient issue with the sigmoid activation function used in the experiment are highlighted, suggesting the exploration of alternative activation functions for more efficient training.

A Bio-inspired Intelligent Industrial Robot Control System (BIIRCS) is proposed in [7], using deep learning methods for effective control of robots. The system incorporates a bio-inspired neural network to model complex environments and guide a team of robots for coverage tasks, imitating the human brain’s ability to process visual information and adapt to dynamic environments.

In [8], training of convolutional neural networks is conducted on the MobileNetV2, ResNet50, and DenseNet121 architectures, with the addition of a Squeeze-and-Excitation (SE) block also for visual information processing and robotic control. The capsular convolutional neural network (CapsNet) is analyzed in [9] for robotic control, where the authors developed two modifications of 1D-CapsNet and Windowed Fourier Transform (WFT)—2D-CapsNet.

Despite attempting to mimic the functioning of biological neurons, ANN-based implementations are either slow or consume significant amounts of energy compared to the human brain. ANN are designed to address problems with well-structured data, typically using standard analog representations for neuronal activity. They lack the ability to bridge the gap between biological neuronal encoding and movement coordination, thus foregoing any attempt to establish biological analogies [10].

Controllers inspired by the functioning of biologic systems have also been developed, as presented in [11,12], where the optimization of a neuroendocrine PID controller based on the Adaptive Safe Experimentation Dynamics (ASED) method is proposed. The neuroendocrine PID controller used is inspired by the ultra-short feedback regulation mechanism of the endocrine system in the human body, achieving a combination of a data-driven mechanism, which utilizes runtime data to optimize controller parameters, and a bio-inspired tuning algorithm, serving as a guide for optimizing the data-driven controller.

In [13], an intelligent controller called the Brain Emotional Learning-Based Intelligent Controller (BELBIC) is proposed. Inspired by brain emotional learning, the BELBIC is designed to address the lack of knowledge regarding nonlinearity and uncertainties in cable-driven robot models. It is based on a computational model comprising four main subsystems: Amygdala, Orbitofrontal Cortex, Sensory Cortex, and Thalamus. Validated on a cable-driven parallel robot, the BELBIC demonstrates effectiveness in trajectory tracking and maintaining positive cable tensions, eliminating the need for calculating the Jacobian matrix and forward kinematics in the feedback loop.

With recent advances in artificial intelligence, new perspectives have emerged in control strategies, aiming to achieve performance more analogous to the human brain [14]. In biological systems, information is processed using relatively small populations of spikes and their precise synchronization, sufficient for driving learning and behavior.

Therefore, spiking neural networks (SNN), regarded as the third generation of neural networks, offer a promising solution to the control challenges in robotics by closely emulating the operational mechanisms of the brain with enhanced biological accuracy, making them the most realistic models of brain function to date [15].

SNN employ pulse coding mechanisms, which allow them to incorporate spatio–temporal information, enabling accurate time modeling and acquiring information with greater accuracy. In addition, they can encode large amounts of information in the relative timing between spikes, leading to the possibility of faster and more efficient implementations [16].

Due to the use of discrete events in processing, SNN compute a single response across multiple time steps, making them less efficient on standard synchronous computer hardware but potentially more effective on specialized neuromorphic hardware [17]. This specialized hardware, comprising asynchronous and event-driven circuits, guides the design of building blocks for hardware solutions, particularly advantageous for robotic platforms [18].

The most appealing features of these networks for robotic applications are their adaptability, self-learning capabilities, and low power consumption. Additionally, they offer faster processing speed and quick response to external stimuli, enabling greater autonomy and swiftness in robots.

Similar to how neural networks in the brain acquire information through electrical impulses or action potentials, adjusting synaptic strengths or the weight of interconnections based on the duration of these action potentials, SNN operate using discrete time-based events. These events are represented as binary electrical spikes, which convey information throughout the network via a spikes train.

The generation or absence of these spikes is determined by the conditions established in the employed neuron model, which typically possesses a set of tunable parameters (transmission delays, synaptic weights, post-spike response and stimulation threshold). The operation of the network depends on these parameters. Several spike-based neuron models are available, and the choice among them is typically influenced by a trade-off between their biological realism and their computational efficiency.

Learning mechanisms, similar to traditional ANN (Artificial Neural Networks), can be categorized into unsupervised learning, supervised learning, and reinforcement learning. Among these, spike-timing-dependent Plasticity (STDP) is one of the most commonly used mechanisms, closely related in biology to Hebb’s postulate: “Neurons that fire together, wire together” [19].

Within the scope of this study, the essential characteristics and components of SNN are addressed. A review of the current state of knowledge in this field is conducted, examining the neuron models and learning mechanisms employed in SNN development. Furthermore, various implementations of SNN in robot control applications are analyzed. Subsequently, a novel spiking PID controller is designed and simulated, employing the NEF philosophy. Finally, a comparative evaluation of the results obtained with the designed controller is established in relation to the performance achieved by a conventional PID, a fuzzy controller, and an ANFIS (adaptive neuro-fuzzy inference system) controller.

The major contributions of this work are described below:

- This research introduces and explores the application of spiking neural networks in robotic controllers, providing valuable insights into their efficacy when compared to conventional controllers;
- Leveraging NEF principles, this research showcases the design and simulation of a novel spiking PID controller for a 3-DoF robotic arm, highlighting NEF’s adaptability in developing bio-inspired control systems;
- Competitive performance was obtained, surpassing a fuzzy controller by 5% in terms of the ITAE index and a conventional PID controller by 6% in the ITAE index and 30% in RMSE performance;
- Emphasizing the substantial potential of SNN within the NEF, this study positions SNN as a promising and versatile choice for advanced robotic applications. This contributes to the domain of industrial robotics through a quantitative analysis, offering a clear understanding of the controller’s practical effectiveness.

The structure of the work comprises Section 2, where the state of the art in the use of SNN for robotic control applications is analyzed. Section 3 covers the fundamentals of SNN, exploring the most commonly used neuron models and learning mechanisms. NEF is examined in Section 4. The development of the controller and the obtained results are addressed in Section 5. Section 6 corresponds to the conclusions.

## 2. SNN in Robotic Control

The challenge of controlling an n-DOF (degrees of freedom) robotic arm is closely tied to establishing the relationship between the robot’s configuration space and the Cartesian space. This relationship enables the positioning of the end effector through the corresponding joint motions, thereby facilitating the execution of the desired movement.

The inverse robot kinematics problem is usually addressed through numerical optimization, as a given end effector position can be achieved through multiple joint configurations. Recent studies suggest the use of SNN to approximate the inverse kinematic model of the robot and execute the control action.

In [20], the design of a three-layer SNN (encoding, learning, and readout) is examined, which can estimate the kinematic properties of a 4-DoF robotic arm. The network takes as inputs the initial positions of the joints and the displacement of the end effector’s relative position. The learning is achieved using a supervised learning rule.

A similar approach is employed in [21] to solve the inverse kinematics of a 6-DoF robot. In this case, an additional signal is introduced to inhibit model learning once a certain precision threshold is reached. The authors implement online learning, where weight adaptation takes place in real time, which proves advantageous in dynamic and disturbed environments. Furthermore, the authors incorporate a PID controller, also designed using SNN.

When dealing with highly redundant robots and those with numerous degrees of freedom, finding the solution to inverse kinematics becomes an even more complex task, as demonstrated by the works presented in [22,23]. A variant of a recurrent SNN, known as LSNN (Long Short-Term Memory spiking neural network), trainable through error gradients, was introduced in [24,25]. These works demonstrated the competitiveness of LSNN compared to other similar second-generation networks like LSTM (Long Short-Term Memory). In [26], the authors propose the use of this recurrent SNN to learn the kinematic model and exert control over a trunk-type robotic arm. They showcased its capability to effectively control such robots with up to 25-DoF with nearly millimeter precision.

Because communication in SNN occurs through spikes, which are non-differential signals, the well-known backpropagation mechanism is not applicable for training these networks [27]. For this reason, some studies focus on obtaining an SNN by converting a pre-trained second-generation network. This practice was appealing as it allowed the advantages in terms of latency and efficiency offered by SNN, while the network was trained with established and efficient methodologies. The works presented in [28,29] employ this approach to perform real-time image classification more efficiently by running the network on specialized neuromorphic hardware, despite a slight decrease in accuracy during the conversion process.

When developing control systems based on SNN, some authors have argued that it has not been formally proven that these third-generation networks offer a substantial improvement in accuracy compared to their predecessors. However, their superiority in hardware implementations in terms of energy efficiency has been demonstrated. For some researchers, this reason alone makes the study of the applicability of SNN in control tasks of significant importance [20].

Indeed, the processing and information representation in SNN are fundamentally different from those employed by conventional neural networks. Consequently, trying to use the same learning mechanisms not only fails to harness the full potential of SNN but might also limit the results to being merely comparable to traditional networks rather than surpassing them. For instance, many gradient descent algorithms, such as mapping-based approaches, often fail to adequately account for the temporal aspect of calculations in SNN [30].

For decades, the true potential of these networks has not been realized in practice. However, recent advancements suggest a shift in this trend, with promising progress in neuromorphic hardware and methodologies [26]. New strategies have emerged to tackle the SNN training challenge, including evolutionary algorithms, Liquid State Machines (LSM), and the neuroscience-inspired STDP rule. These approaches unlock a realm of possibilities for deploying and refining SNN architectures by harnessing their intrinsic characteristics.

An example of the advantages that the use of SNN can provide is in torque control. Torque control is concerned with the dynamics of a robot, which encompasses its changes over time. As a result, the utilization of temporal encoding is well suited for capturing the progression of sensorimotor signals and for effectively controlling motion. This makes them an interesting solution for adaptive robot control [10].

## 3. SNN Fundamentals

In neural connections, each neuron connects with around 10,000 others, processes information continuously, and consumes minimal energy in comparison to the millions of existing neurons. It self-organizes and reconfigures over time. Replicating this behavior in an artificial system presents a highly complex endeavor. This complexity persists even when employing simplifications that only capture a fraction of the biological richness.

Over time, models and algorithms have been developed to, in some way, attempt to mimic this neuronal behavior. These developments have evolved in conjunction with advancements in computing resources and neuroscience discoveries.

The first generation of neural networks exclusively dealt with binary signals using a threshold function as their activation function. Given their proven capacity to effectively approximate analog functions with a high degree of accuracy, these networks have found extensive application as powerful tools for information processing.

In the second generation, nonlinear and continuous activation functions began to be used, allowing for the representation of analog values, typically scaled to a small numerical range. These neurons are more powerful than their predecessors because they can perform the same function but with significantly fewer neurons. Due to the demonstrated ability of these networks to approximate analog functions arbitrarily well, they have been widely used in machine learning applications.

The third generation enables the use of temporal information in communication and computation, bringing it closer to the functioning of biological neurons. Space–time information is captured by the SNN, encoded in spikes. The transmission of event timing with remarkable precision and accuracy is a notable attribute of these networks’ spikes. SNN offer distinct advantages and heightened biological plausibility, making them well suited for applications in robotics. They also contribute to the development of improved tools for analyzing brain functions [31].

### 3.1. Biological Neurons

**Figure 1:**
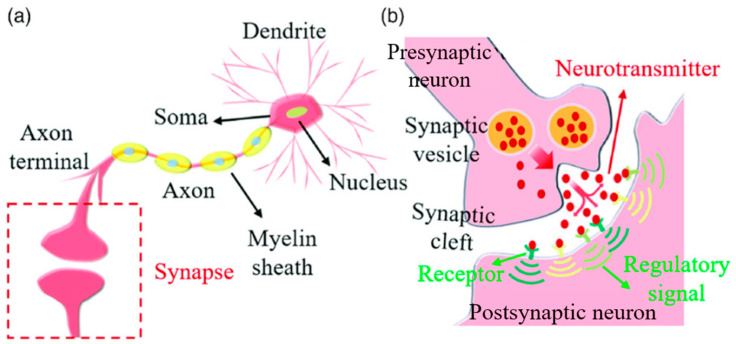
Biological neurons and synapses. (**a**) Structure and components of neurons. (**b**) Schematic diagram of a synapse [34].

In the human nervous system, around 86 billion neurons serve as fundamental operational units [32], transmitting electrochemical signals known as action potentials [33]. Neurons have a complex structure, shown in Figure 1a, including the soma (cell body) for information processing, dendrites for receiving impulses, and axons for transmitting impulses. The neuron’s membrane potential increases through dendritic input, leading to an action potential or spike [16]. Following a spike, there is a refractory period, preventing immediate firing. The generated spike travels through the axon to other neurons.

Action potentials arriving at axon terminals initiate the release of neurotransmitters into the synaptic cleft, binding to receptors on the postsynaptic neuron’s membrane and altering its potential. Neurotransmitters can have excitatory or inhibitory effects, facilitating information transmission. This neuronal connection, known as a synapse, is a fundamental and intricate element of neural function. This phenomenon is illustrated in Figure 1b.

While the exact mechanism by which biological neural systems respond to external stimuli remains uncertain, it is widely accepted that synapses either strengthen or weaken, adapting the response to stimuli based on the outcome they achieve. In this process of strengthening and weakening, synapses, in turn, induce the inhibition of certain neurons in favor of others, ensuring the response is as closely aligned to the received stimuli as possible.

### 3.2. Neuron Models

Due to the complexity of the dynamics in live neuronal cells resulting from the exchange of ions, their precise implementation in computationally intensive models is highly challenging. Nevertheless, the fundamental elements of their operation, such as dynamics, propagation, and plasticity, can be successfully modeled using mathematical descriptions.

Various models of neurons with different levels of complexity and biological plausibility have been proposed. Typically, the selection of a specific model involves a trade-off between these two elements, depending on the intended application. In [35], a review of spike neuron models is conducted, taking these aspects into account.

The Hodgkin–Huxley model [36,37] is a classical spiking neuron model that successfully captures the detailed dynamics of ion channels in real neurons. A comprehensive computational model of a biological neuron, like the Hodgkin–Huxley model, is impractical for simulation development due to its strong computational demands. To address this challenge, various more efficient models have been developed [38]. These models are simpler but can still capture the essence of the dynamics of real neurons. Some of these models, along with their key characteristics, are presented in Table 1.

### 3.3. Learning Rules

Approaches to training SNN can be broadly categorized into three groups: supervised learning, which involves techniques like gradient descent and spike backpropagation; unsupervised learning, employing local synaptic learning rules, such as spike-timing-dependent plasticity; and reinforcement learning, which relies on reward or error signals and uses reward-modulated plasticity for training [33].

The first supervised learning algorithm for spiking neurons, SpikeProp, utilizes backpropagation and gradient descent [43]. SpikeProp formulates an error function based on the variance between the intended and observed output spike times, constructed using the least squares error method [44]. Another approach is the SuperSpike algorithm [45], a nonlinear voltage-based learning rule employing gradient descent grounded in the membrane potential of neurons [46]. Tempotron, an online supervised learning rule, is designed for binary classification of multi-neuronal spike patterns. It relies on gradients and adapts synaptic weights to fire appropriately in response to spike patterns from various categories [47], representing a biologically plausible mechanism [48].

Biological systems constantly adapt, can learn from their mistakes, and can recognize new patterns they have never seen before. This type of learning is not naturally captured by backpropagation techniques. The backpropagation algorithm is effective when dealing with many inputs and outputs and when the process occurring between them is not well known. However, when there is a clear idea of what needs to be represented, then using that information can be useful and beneficial.

Prescribed Error Sensitivity (PES) is an online supervised learning algorithm tailored for real-time adaptive control. This approach improves a function by minimizing an externally provided error signal [49]. It is frequently used in conjunction with the NEF.

Liquid State Machine (LSM) is a widely used algorithm in SNN [30]. In this paradigm, a sparsely connected recurrent SNN functions as a dynamic “liquid” or “reservoir.” The reservoir, constructed stochastically, maintains input distinctiveness and transient memory. A readout component, often employing linear regression, interprets the reservoir’s output.

Evolutionary approaches have also been used to train or design SNN [30]. These approaches are advantageous due to their independence from differentiable activation functions and network configurations. They provide flexibility for adapting and modifying network features, but this flexibility may result in slower convergence compared to alternative training methods.

Spike-timing-dependent plasticity (STDP) is an unsupervised Hebbian learning method that adjusts the connection strength between neurons by considering the relative spike timing [34]. When the presynaptic spike arrives before a postsynaptic spike, the synaptic weight increases; this phenomenon is known as Long-Term Potentiation (LTP). Conversely, if the synaptic spike arrives after the postsynaptic spike, it results in Long-Term Depression (LTD), causing a decrease in synaptic weight.

## 4. Neural Engineering Framework

The Neural Engineering Framework (NEF) is a comprehensive methodology for developing large-scale, biologically plausible cognitive models [50]. It ensures a globally optimal approximation of dynamic equations, balancing high-level abstraction with preservation of fundamental behavioral aspects. Unlike frameworks for learning from input–output data, the NEF constructs a spiking neural network with a known transform through an optimization procedure [51].

The NEF translates neural activity into a vector space representation, implementing ordinary differential equations (ODE) [52]. The capability to implement ODE makes it suitable for control theory algorithms, with real-time adjustment of connection weights.

The NEF handles recurrent connections for complex dynamical systems and integrates error-based learning rules for online adaptation [53]. Biological constraints are incorporated by defining specific neuronal characteristics, allowing the evaluation of algorithm feasibility by comparing them with data at various levels.

The NEF consists of three fundamental principles: representation, transformation, and dynamics.

The principle of representation outlines how the NEF represents information using patterns of neuronal activity (in the form of time-varying vectors of real numbers) in a neural ensemble through the combination of nonlinear encoding and weighted linear decoding.

Following this principle, input signals are encoded into populations of neurons using specific tuning curves, which describe the activation of each neuron in response to the input signal. Each neuron i in the ensemble has an encoding vector (encoder) $e_{i}$, interpreted as the preferred direction vector of the neuron, meaning the vector for which the neuron will fire most intensely.

Starting from the premise that the NEF establishes the input current to a neuron governed by a linear function of the represented value, neuronal activity for an input value x is calculated using Equation (1), where G is the neuronal nonlinearity (dependent on the chosen neuron model), $\alpha_{i}$ is a gain parameter, and $I_{i}^{bias}$ is the constant background current for the neuron.

(1) $$a_{i}=G_{i}(\alpha_{i}e_{i}\cdot x+I_{i}^{bias})$$

Subsequently, applying the same principle, the spike activity generated in an ensemble is decoded to obtain the represented value. The estimation of this value relies on the postsynaptic activity generated after receiving a spike through the synapse. This activity is obtained by applying a linear filter to the spike train according to Equation (2), where $a_{i}$ is the activity of neuron i, $h_{i}$ is the spike response function (typically a decaying exponential weighted by a time constant), * is the convolution operator, and $\delta_{j}$ is the spike train generated in response to the input signal x, with spike times indexed by j.

(2) $$a_{i}\left(x\right)=h_{i}\left(t\right)*\delta_{j}(t-t_{j}(x))$$

This enables the definition of the decoding operation as a linear sum of the neuronal activities of that population, according to Equation (3), where $\hat{x}$ represents the estimation of the original input signal, N is the number of neurons in the population, and $d_{i}$ is the decoder. The least squares minimization is the most commonly used method to determine the set of decoding weights $d_{i}$ as it is necessary to minimize the difference between the represented value x and its estimation.

(3) $$\hat{x}=\sum_{i}^{N}a_{i}(x)d_{i}$$

The principle of transformation explains how operations and transformations between neural ensembles are implemented to construct the neural network and execute functions through their connections.

When a neuron produces a spike, it releases a neurotransmitter through the synapse, typically resulting in the transmission of a certain amount of current to the postsynaptic neuron. Many factors influence the amplitude of this current, and in the NEF, these factors are encapsulated in a scalar connection weight representing the strength of the connection between two neurons.

According to this principle, the connection weights between ensembles are computed as the product of the decoding weights for the function in the first ensemble $d_{i}$, the encoding weights for the second ensemble $e_{j}$, and some linear transformation, as defined in Equation (4). This principle also allows for the addition of values simply by introducing two inputs into the same group of neurons.

(4) $$\omega_{ij}=f(d_{i}\cdot e_{j})$$

The principle of dynamics establishes that neural representations can be viewed as state variables in a dynamic system (linear or nonlinear). These dynamic systems are built through recurrent connections, which can be computed using the second principle.

### Nengo

Nengo Brain Maker is a powerful neural simulator based on the NEF, designed for building large-scale models of interconnected neural systems. Utilizing neural ensembles to represent information, Nengo acts as a “neural compiler,” translating high-level functional models into detailed low-level neural networks [54]. The Python package comprises essential objects like Ensemble, Node, Connection, Probe, and Network, while NengoGUI, an interactive web tool, aids in model visualization.

This tool provides versatility in defining parameters within neuron ensembles, allowing adjustments to firing rates, representational radius, and intercepts. It enables the incorporation of synaptic time constants, anatomical limitations, and alignment with neuroscientific evidence in the model design [55]. Nengo supports various biologically plausible learning rules for connection weight adjustments, with the PES rule being commonly used.

In comparison to other neural simulators, Nengo stands out for its theoretical framework with high biological realism. It played a crucial role in developing Spaun 2.0, the largest functional brain model, making Nengo truly unique in its class. Notably, Nengo boasts a speed advantage by storing only elements of the connection weight matrix, ensuring flexibility, scalability, and robustness.

## 5. Spiking PID Controller

In this section, we present the development of a PID (Proportional–Integral–Derivative) controller using spiking neurons under the NEF philosophy. The structure and operation of the controller are described, as well as the simulation environment used. An analysis of the controller’s behavior is performed using the graphs obtained in the trajectory tracking and performance indices.

### 5.1. Description of the System under Study

For this study, the subject of control is a 3-DoF robotic arm, characterized by the Denavit–Hartenberg parameters detailed in Table 2, with $d_{1}=0.352\;m$, $a_{2}=0.36\;m$, and $a_{3}=0.445\;m$. The dynamic model of the robotic arm was derived employing the Lagrange–Euler formulation, as specified in Equation (5).

(5) $$\tau =M\left(q\right)\ddot{q}+C\left(q,\dot{q}\right)+G\left(q\right)+F(\dot{q})$$

where M(q) stands for the inertia matrix, $C\left(q,\dot{q}\right)$ signifies the matrix encompassing Coriolis terms, $G\left(q\right)$ represents the vector denoting gravitational torques acting on the robot, and $F(\dot{q})$ pertains to the vector of frictional forces. τ is utilized for the vector of generalized forces and $q,\dot{q},$ and $\ddot{q}\;$represent the components of the position vector, velocity, and acceleration of the joints, respectively. The dynamic model of the 3-DoF manipulator is expressed by Equations (6) through (25) with the corresponding dynamical parameter values detailed in Table 3. The mathematical model of the robot was adopted from [56].

(6) $$M(q)=\left[\begin{array}{ccc} m_{11} & m_{12} & m_{13}\\ m_{21} & m_{22} & m_{23}\\ m_{31} & m_{32} & m_{33} \end{array}\right]$$

(7) $$m_{11}={(m}_{3}{l_{c3}}^{2}+I_{y3}){c_{3}}^{2}+2a_{2}m_{3}l_{c3}c_{2}c_{3}+(m_{2}{l_{c2}}^{2}+{a_{2}}^{2}m_{3}I_{y2}){c_{2}}^{2}$$

(8) $$m_{12}=m_{13}=m_{21}=m_{31}=0$$

(9) $$m_{22}=2a_{2}m_{3}l_{c3}c_{23}+m_{3}{l_{c3}}^{2}+m_{2}{l_{c2}}^{2}+{a_{2}}^{2}m_{3}+I_{z3}+I_{z2}$$

(10) $$m_{23}=m_{32}=a_{2}m_{3}l_{c3}c_{23}+m_{3}{l_{c3}}^{2}+I_{z3}$$

(11) $$m_{33}=m_{3}{l_{c3}}^{2}+I_{z3}$$

(12) $$I_{y2}=I_{z2}=\frac{m_{2}{a_{2}}^{2}}{12}$$

(13) $$I_{y3}=I_{z3}=\frac{m_{3}{a_{3}}^{2}}{12}$$

(14) $$C(q,\dot{q})=\frac{1}{2}\left[\begin{array}{ccc} C_{11} & C_{12} & C_{13}\\ C_{21} & C_{22} & C_{23}\\ C_{31} & C_{32} & C_{33} \end{array}\right]$$

(15) $$\begin{array}{c} C_{11}=-\{m_{2}l_{c2}^{2}sen\left(2\theta_{2}\right)\\ \;\;\;\;\;\;\;+m_{3}\left(a_{2}^{2}sen\left(2\theta_{2}\right)+l_{c3}^{2}sen\left(2\theta_{2}+2\theta_{3}\right)+a_{2}l_{c3}sen\left(2\theta_{2}+\theta_{3}\right)\right)\}\dot{\theta_{2}}\\ \;\;\;\;\;\;\;\;-\{m_{3}l_{c3}(l_{c3}sen\left(2\theta_{2}+2\theta_{3}\right)+2a_{2}sen\left(2\theta_{2}+\theta_{3}\right)+2a_{2}sen\left(\theta_{3}\right))\}\dot{\theta_{3}} \end{array}$$

(16) $$\begin{array}{c} C_{12}=-\{m_{2}l_{c2}^{2}sen\left(2\theta_{2}\right)+m_{3}(a_{2}^{2}sen\left(2\theta_{2}\right)+l_{c3}^{2}sen\left(2\theta_{2}+2\theta_{3}\right)\\ +a_{2}l_{c3}sen\left(2\theta_{2}+\theta_{3}\right))\}\;\dot{\theta_{1}} \end{array}$$

(17) $$C_{13}=-\{m_{3}l_{c3}(l_{c3}sen\left(2\theta_{2}+2\theta_{3}\right)+2a_{2}sen\left(2\theta_{2}+\theta_{3}\right)+2a_{2}sen\left(\theta_{3}\right))\}\dot{\theta_{1}}$$

(18) $$\begin{array}{c} C_{21}=-\{m_{2}l_{c2}^{2}sen\left(2\theta_{2}\right)+m_{3}(a_{2}^{2}sen\left(2\theta_{2}\right)+l_{c3}^{2}sen\left(2\theta_{2}+2\theta_{3}\right)\\ +a_{2}l_{c3}sen\left(2\theta_{2}+\theta_{3}\right))\}\;\dot{\theta_{1}} \end{array}$$

(19) $$C_{22}=-\frac{m_{3}a_{2}l_{c3}s_{23}}{2}\dot{\theta_{3}}$$

(20) $$C_{23}=-\frac{m_{3}a_{2}l_{c3}s_{23}}{2}(\dot{\theta_{2}}+\dot{\theta_{3}})$$

(21) $$C_{31}=-\{m_{3}l_{c3}(l_{c3}sen\left(2\theta_{2}+2\theta_{3}\right)+2a_{2}sen\left(2\theta_{2}+\theta_{3}\right)+2a_{2}sen\left(\theta_{3}\right))\}\dot{\theta_{1}}$$

(22) $$C_{32}=\frac{m_{3}a_{2}l_{c3}s_{23}}{2}\dot{\theta_{2}}$$

(23) $$C_{33}=0$$

(24) $$G\left(q\right)=\left[\begin{array}{c} 0\\ \left(m_{2}l_{c2}c_{2}+m_{3}\left(a_{2}c_{2}+l_{c3}c_{23}\right)\right)g\\ m_{3}l_{c3}c_{23}g \end{array}\right]$$

(25) $$F\left(\dot{q}\right)=\left[\begin{array}{c} F_{v}(\dot{q_{1}})\\ F_{v}(\dot{q_{2}})\\ F_{v}(\dot{q_{3}}) \end{array}\right]=\left[\begin{array}{c} b_{1}\dot{\theta_{1}}\\ b_{2}\dot{\theta_{2}}\\ b_{3}\dot{\theta_{3}} \end{array}\right]$$

### 5.2. Controller Design

**Figure 2:**
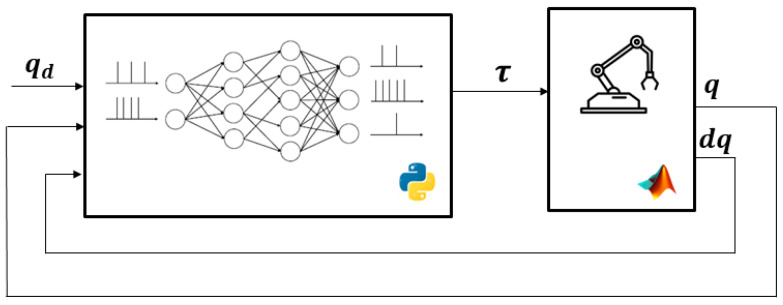
Diagram of the simulation environment used.

Considering the challenge of controlling the joint position of a robotic arm using a bio-inspired system with SNN and the concepts established by the NEF, as well as in analyzing the potentialities and facilities offered by Nengo, a simulation environment is developed based on the diagram shown in Figure 2, where $q_{d}$ represents the desired position and q and dq correspond to the actual positions and velocities of the robot.

The MATLAB/Simulink tool was used to simulate the robot, recreating its kinematic and dynamic characteristics. Additionally, by utilizing MATLAB’s capability to execute Python code, the SNN controller developed using Nengo was integrated.

Given its simplicity and widespread adoption in the industry for robot control, the classic Proportional–Integral–Derivative (PID) controller stands out as the primary choice for implementing industrial control systems.

This controller generates a signal $u\left(t\right)$ that guides the robot joint toward the desired position through continuous error reduction. It consists of three components: the current error value, the derivative of the error (which considers the projected future error value), and the integral of the error (which takes into account past error values). The control signal is calculated by combining these components according to Equation (26), where $k_{p}$, $k_{d}$ and $k_{i}$ represent the proportional, derivative, and integral gains of the controller.

(26) $$u\left(t\right)=k_{p}e\left(t\right)+k_{d}\frac{de(t)}{dt}+k_{i}\int_{0}^{t}e(t)$$

(27) $$e\left(t\right)=q_{d}-q$$

**Figure 3:**
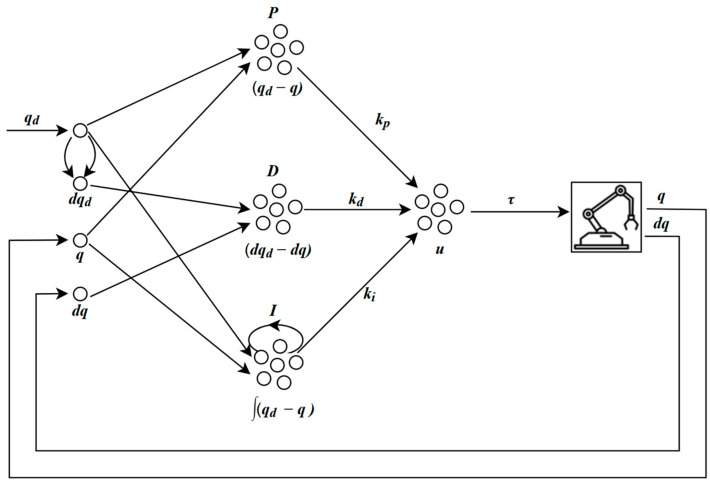
Diagram of the spiking PID controller.

This work proposes simulating PID control using spiking neurons for the calculation of its three components. The configuration of the spiking PID controller simulation in Nengo is illustrated in Figure 3.

Through Nengo nodes, the controller receives the values of $q_{d}$, q, and dq as input and obtains ${dq}_{d}$, corresponding to the desired velocity, by deriving $q_{d}$ through two connections (synapses) with different time scales. These values are encoded to neural activity in the ensembles P, D, and I according to Equation (1), where the error, its derivative, and its integral are obtained, respectively. Subsequently, using Equation (4), in the connection to the u ensemble, a linear transformation is applied to these values, multiplying them by their respective gains $k_{p}$, $k_{d}$, and $k_{i}$. Finally, the u ensemble performs a weighted sum of the three branches of the controller according to Equation (26) and outputs the appropriate torque signal for the robot’s movement.

**Figure 4:**
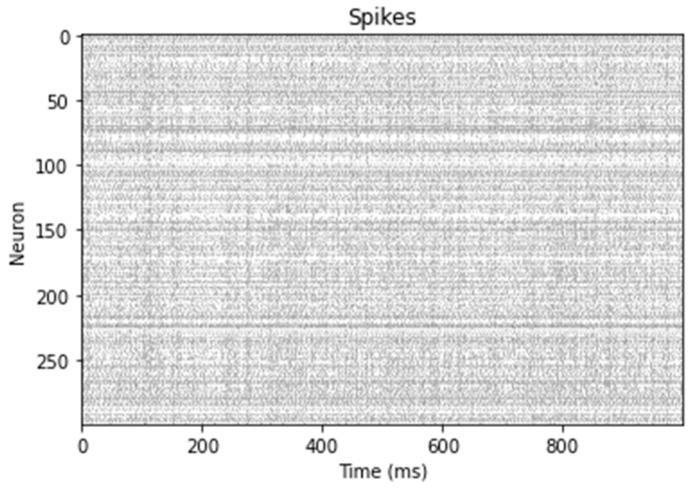
Spike activity of ensemble u during 1 second of simulation.

For each set of input values $q_{d}$, q, and dq, the ensemble u generates a pattern of neuronal activity, meaning a spike pattern, as shown in the Figure 4. This signal is generated in a Python function executed from Simulink using the MATLAB coder.extrinsic. To save the signal generated in the ensemble and use it as a torque signal to control the robot, a Nengo object called Probe is employed (a probe is an object that collects data from the simulation).

The values represented by the neuronal activity of ensemble u are automatically calculated by Nengo according to the Equation (3) and saved in a nengo.Probe. For each input set, the created probe saves the values of u for 1 s in the tau variable; these values are then averaged and returned to MATLAB to be applied as torque to the robot.

**Figure 5:**
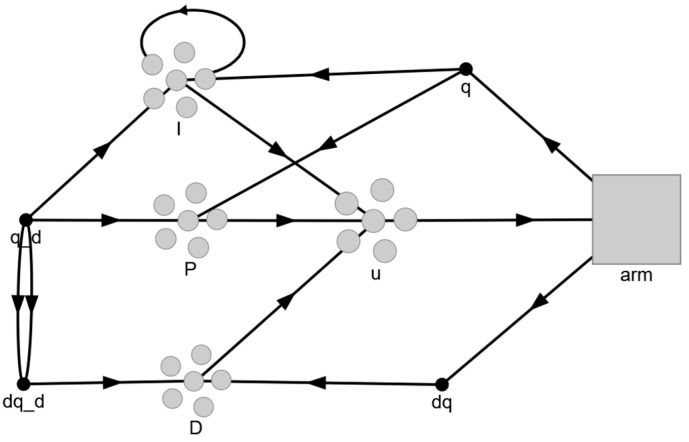
Spiking PID implementation in Nengo.

In this implementation, 300 LIF-type neurons were used for each ensemble, and weight optimization between neural representations was performed during model construction. The Nengo implementation of the spiking PID controller is illustrated in Figure 5.

All the neural operations and transformations for the development of the spiking PID controller are automatically implemented by the Nengo simulator. Hence, the primary advantage of this tool is that it allows one to describe, at a high level of abstraction, the function to be realized. Simply specifying the function you want to compute will result in an SNN that performs that function. Therefore, implementing this controller using these principles is relatively straightforward because the functions to be developed are well known.

A curious aspect of working with these tools is that, regardless of the structure you aim to develop with SNN, even if it involves mapping specific brain regions such as the motor cortex or the cerebellum, in the end, when the model is executed, what you have is a group of individual neurons simulated, each sending spikes to other neurons through synapses with specific weights, just like what occurs in the actual brain.

### 5.3. Results and Discussions

**Figure 6:**
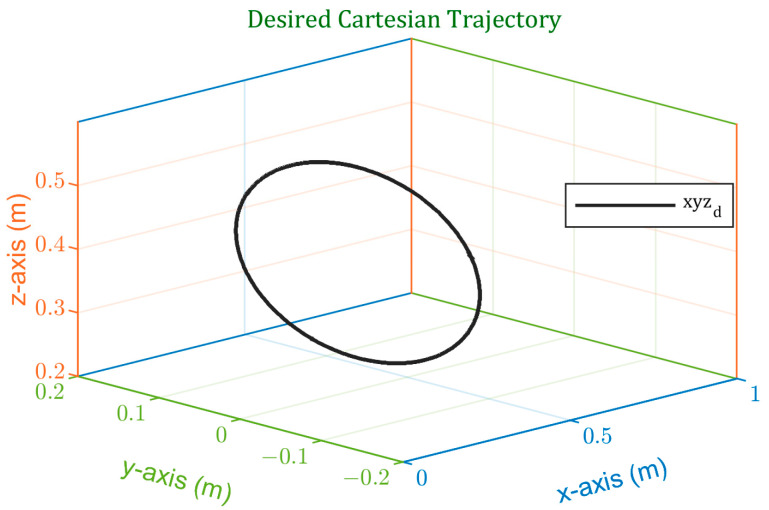
Test trajectory used to evaluate the performance of the spiking PID controller.

To evaluate the performance of the designed spiking PID controller, a circular trajectory was used with sinusoidal position and velocity profiles. The employed trajectory is defined by Equation (28) and is depicted in Figure 6.

(28) $$\left\{\begin{array}{c} x=0.352\\ y=0.15\;\sin\left(2t\right)\\ z=0.15\;\cos\left(2t\right)+0.4 \end{array}\right.$$

**Figure 7:**
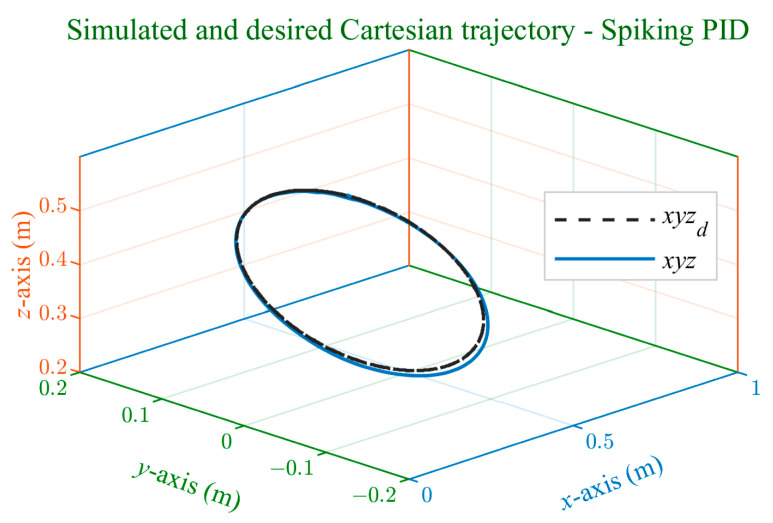
Simulated and desired Cartesian trajectory using spiking PID controller.

**Figure 8:**
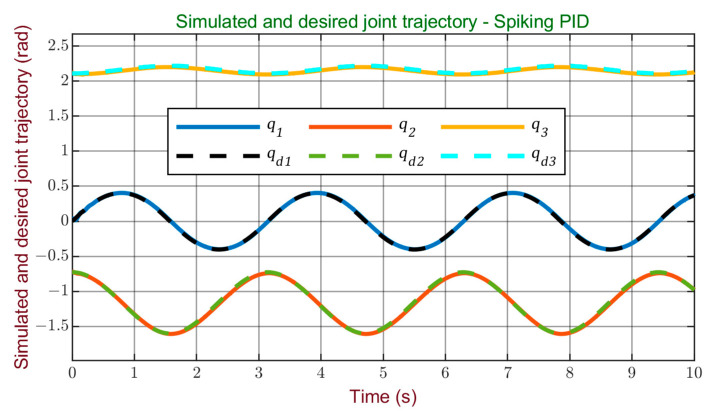
Simulated and desired joint trajectory using spiking PID controller.

The trajectory tracking graphs, depicted in Figure 7 for the Cartesian trajectory and Figure 8 for the joint trajectory, illustrate the controller’s effectiveness. The robot consistently maintains accurate tracking of the reference trajectory, demonstrating smooth performance without overshoots or abrupt deviations.

**Figure 9:**
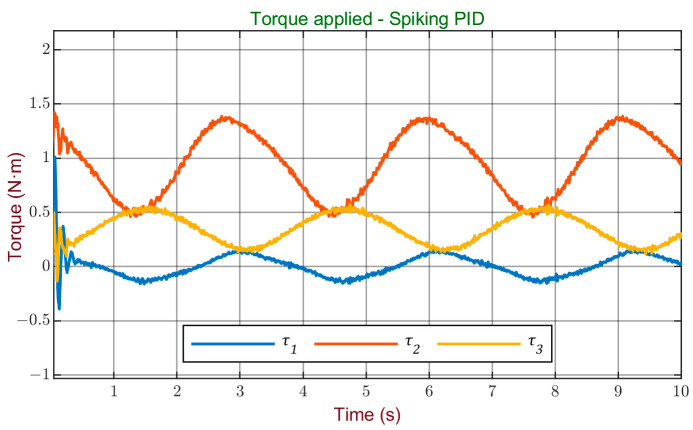
Torque signal applied to the robot using spiking PID controller.

The torque signal obtained from the controller is shown in Figure 9, where a smooth variation can be observed, without abrupt changes and without the presence of signal saturations.

**Figure 10:**
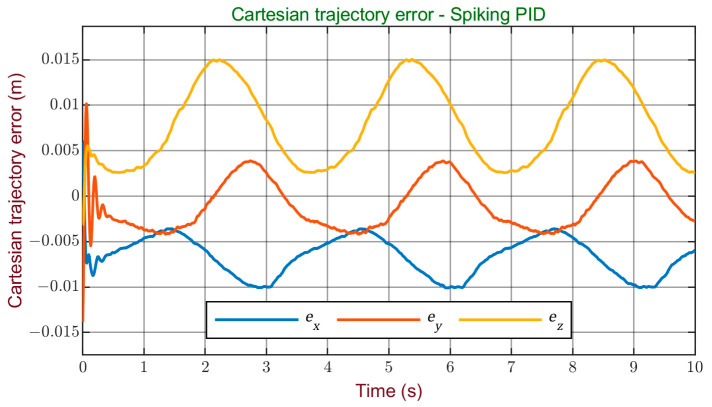
Cartesian trajectory error with the spiking PID controller.

**Figure 11:**
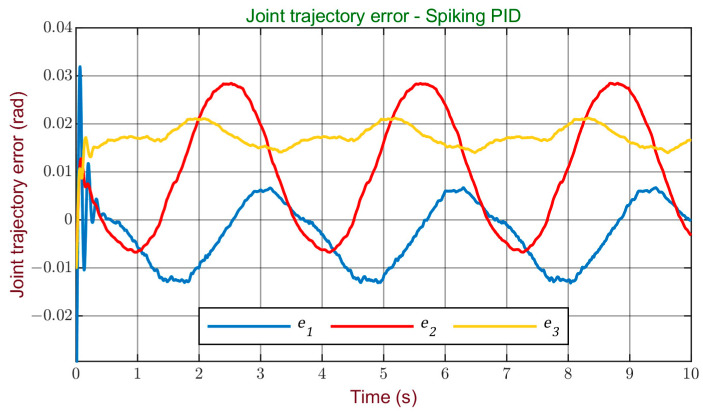
Joint trajectory error with the spiking PID controller.

Figure 10 and Figure 11 display the errors obtained during the simulation for the Cartesian and joint trajectories, offering a visual representation of the system’s performance in terms of tracking accuracy. It can be observed that, although further enhancements are possible, the errors obtained are minimal. Therefore, it can be stated that a satisfactory performance was achieved with this controller, reaching a high level of accuracy in reference tracking.

The spiking PID controller’s performance was evaluated through the following performance indices: residual mean square error (RMSE), assessing the accuracy of the control model, and the integral of time-weighted absolute error (ITAE), measuring the steady-state error in the initial response. Equations (29) and (30) detail the expressions for calculating these indices, providing a comprehensive assessment of the controller’s effectiveness.

(29) $$RMSE=\sqrt{\frac{1}{n}\sum_{i=1}^{n}{e_{i}}^{2}}\;\;\;\;\;\;\;\;\;\;[rad]$$

(30) $$ITAE=\int_{0}^{\infty }t\left|e(t)\right|dt\;\;\;\;\;\;\;\;\;\;[rad\cdot s]$$

For a quantitative performance analysis, the metrics obtained with the spiking PID controller were compared with those of a conventional PID controller using identical gains. Additionally, comparisons were made with a previously developed fuzzy controller and an ANFIS (adaptive neuro-fuzzy inference system) controller designed for this plant, as detailed in [56]. The evaluation encompassed both Cartesian and joint trajectories, and the corresponding metrics are presented in Table 4 and Table 5.

**Figure 12:**
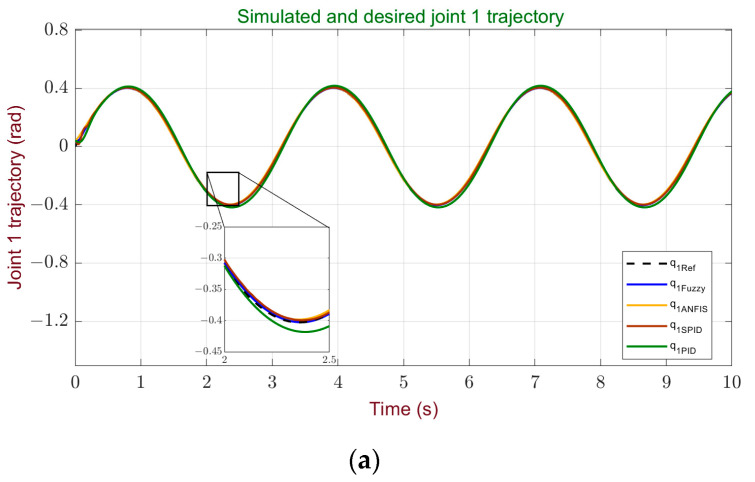

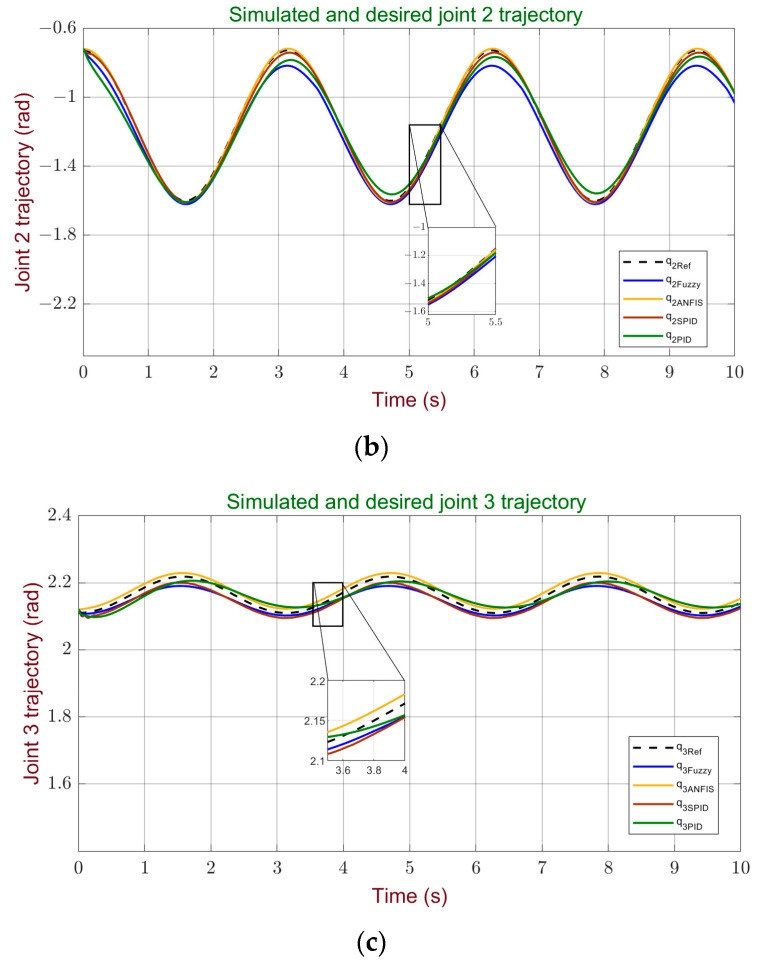
Joint trajectory performance comparison of the controllers: (**a**) performance comparison for joint 1; (**b**) performance comparison for joint 2; (**c**) performance comparison for joint 3.

In general, the obtained indices are good and quite close to those achieved by more traditional controllers. This conclusion is supported by the graphical representation in Figure 12, allowing for a comparative analysis of the controllers’ response through the tracking curves of each robot joint.

The spiking PID controller demonstrates competitive performance with good accuracy and rapid error correction. Analysis of Table 4 and Table 5 shows that it outperforms the fuzzy controller by 5% in terms of the ITAE index.

The designed spiking PID controller shows improvements of 6% in the ITAE index and 30% in RMSE performance compared to the conventional PID controller, despite being designed with the same gains. This improvement is attributed to the spiking PID controller’s ability to maintain a more faithful representation of temporal patterns and system dynamics, leveraging the biological similarity of neurons and synaptic connections.

The approach used in Nengo enables advanced spatio–temporal processing, capturing complex relationships between input and output signals. The distributed representation of information in SNN facilitates efficient signal encoding, resulting in increased adaptability and precision in generating control signals.

While the spiking PID controller does not surpass the performance of the ANFIS controller, the result is considered satisfactory, considering that it is in an early stage of research. Additionally, this design incorporates some improvements inherent to SNN but retains certain intrinsic characteristics of the PID controller, limiting its adaptability to complex nonlinear systems, especially those with dynamic and variable behaviors. The manual tuning of PID gains, although crucial, may not be sufficient to address all operational conditions. Furthermore, the complexity of the robot and assigned tasks can significantly influence the relative performance of controllers. The ANFIS, being an adaptive neuro-fuzzy inference system, proves to be more adept at modeling and adapting to the specific nonlinearities of the system.

## 6. Conclusions

Spiking neural networks (SNN) play a significant role in advancing robotics, particularly in systems with higher degrees of freedom and industrial applications, showcasing a high potential to enhance autonomy and adaptability in dynamic environments. The utilization of the Neural Engineering Framework (NEF) and Nengo provides a powerful, robust, and adaptable approach for SNN-based controllers, as demonstrated by the implementation of a novel spiking Proportional–Integral–Derivative (PID) controller for a robotic arm. The competitive performance achieved by the developed controller is evident through trajectory tracking and error curve analyses, as well as performance indices such as RMSE and ITAE.

This research, representing the initial phase of an ongoing investigation, shows promising results for improved precision compared to conventional controllers. Future research aims to advance SNN-based controllers, focusing on adaptability, precision, and overall performance. The consideration of robotic systems with multiple degrees of freedom constitutes a key aspect of this strategic focus. Furthermore, potential hardware implementations of SNN controllers, utilizing FPGA or specialized neuromorphic hardware, are contemplated for future studies to demonstrate their energy-efficient characteristics and high performance in executing control models for real-time industrial applications.

In light of these findings, this study contributes to the growing body of knowledge on the applicability of SNN in robotics, paving the way for future advancements and practical implementations in industrial and complex robotic scenarios.

## Figures and Tables

**Table 1:** Neuron models.

Model	Formulation	Characteristics
Integrate and Fire (IF)	[32]	Simulates neuron’s behavior rather than its structure. Simplified model of a neuron with fire threshold. Widely recognized and employed.
Leaky Integrate and Fire (LIF)	[32,33,39]	Adds a “leakage” term to the basic IF model. Accounts for the ion movement across the membrane during non-equilibrium states. Minimal computational requirements and simple structure.
Izhikevich	[32,33,40]	Combines biological plausibility and computational efficiency. Adjusting the model’s parameters allows obtaining diverse firing patterns observed in different brain neurons. Viewed as an adaptive quadratic LIF model.
Adaptive Exponential LIF (AdEx)	[33,41,42]	Includes threshold adaptation based on the membrane potential increase. Incorporates an exponential voltage dependency. Captures the adaptability of neurons.

**Table 2:** D–H parameters of the 3-DoF manipulator.

Joint	$a_{i}$	$\alpha_{i}$	$d_{i}$	$\theta_{i}$
1	0	π2	$d_{1}$	$\theta_{1}$
2	$a_{2}$	0	0	$\theta_{2}$
3	$a_{3}$	0	0	$\theta_{3}$

**Table 3:** Parameters considered for the manipulator.

	Link1	Link2	Link3	Units
m	0.5	0.3	0.3	[kg]
l	0.352	0.36	0.445	[m]
$l_{c}$	0.176	0.18	0.22	[m]
$F_{v}$	0.15	0.25	0.25	[N·m·s/rad]

**Table 4:** Performance indices for Cartesian trajectory.

	ITAE				RMSE			
	PID	Fuzzy	ANFIS	Spiking PID	PID	Fuzzy	ANFIS	Spiking PID
x	0.1577	0.4673	0.1923	0.3449	0.0064	0.0087	0.0039	0.0067
y	0.3054	0.1211	0.0825	0.1253	0.0068	0.0030	0.0023	0.0033
z	0.4505	1.17	0.1052	0.4158	0.0153	0.0217	0.0023	0.0089

**Table 5:** Performance indices for joint trajectory.

	ITAE				RMSE			
	PID	Fuzzy	ANFIS	Spiking PID	PID	Fuzzy	ANFIS	Spiking PID
$q_{1}$	0.8909	0.1367	0.1213	0.2896	0.0189	0.0054	0.0047	0.0087
$q_{2}$	1.569	2.573	0.3517	0.6893	0.0412	0.0482	0.0079	0.0155
$q_{3}$	0.6513	0.7583	0.4471	0.8631	0.0142	0.0174	0.0089	0.0169

## Data Availability

All research data is included in the paper.

## References

[1] Kalsoom T., Ramzan N., Ahmed S., Ur-Rehman M. (2020). Advances in Sensor Technologies in the Era of Smart Factory and Industry 4.0. Sensors.

[2] Indri M., Grau A., Ruderman M. (2018). Guest Editorial Special Section on Recent Trends and Developments in Industry 4.0 Motivated Robotic Solutions. IEEE Trans. Ind. Inform..

[3] Jones A., Gandhi V., Mahiddine A.Y., Huyck C. (2023). Bridging Neuroscience and Robotics: Spiking Neural Networks in Action. Sensors.

[4] Xu K., Wang Z. (2023). The Design of a Neural Network-Based Adaptive Control Method for Robotic Arm Trajectory Tracking. Neural Comput. Appl..

[5] Guo J., Nguyen H.-T., Liu C., Cheah C.C. (2023). Convolutional Neural Network-Based Robot Control for an Eye-in-Hand Camera. IEEE Trans. Syst. Man Cybern. Syst..

[6] Xu P. Neural Network Based Self-Tuning PID Controller. Proceedings of the 2022 2nd International Conference on Algorithms, High Performance Computing and Artificial Intelligence (AHPCAI).

[7] Guan J., Su Y., Su L., Sivaparthipan C.B., Muthu B. (2021). Bio-Inspired Algorithms for Industrial Robot Control Using Deep Learning Methods. Sustain. Energy Technol. Assess..

[8] Tsapin D., Pitelinskiy K., Suvorov S., Osipov A., Pleshakova E., Gataullin S. (2023). Machine Learning Methods for the Industrial Robotic Systems Security. J. Comput. Virol. Hacking Tech..

[9] Osipov A., Pleshakova E., Bykov A., Kuzichkin O., Surzhik D., Suvorov S., Gataullin S. (2023). Machine Learning Methods Based on Geophysical Monitoring Data in Low Time Delay Mode for Drilling Optimization. IEEE Access.

[10] Abadía I., Naveros F., Garrido J.A., Ros E., Luque N.R. (2021). On Robot Compliance: A Cerebellar Control Approach. IEEE Trans. Cybern..

[11] Ghazali M.R., Ahmad M.A., Jusof M.F.M., Ismail R.M.T.R. A Data-Driven Neuroendocrine-PID Controller for Underactuated Systems Based on Safe Experimentation Dynamics. Proceedings of the 2018 IEEE 14th International Colloquium on Signal Processing & Its Applications (CSPA).

[12] Bin Ghazali M.R., bin Ahmad M.A., bin Raja Ismail R.M.T. (2022). Adaptive Safe Experimentation Dynamics for Data-Driven Neuroendocrine-PID Control of MIMO Systems. IETE J. Res..

[13] Bajelani M., Ahmad Khalilpour S., Isaac Hosseini M., Taghirad H.D., Cardou P. Brain Emotional Learning Based Intelligent Controller for a Cable-Driven Parallel Robot. Proceedings of the 2021 9th RSI International Conference on Robotics and Mechatronics (ICRoM).

[14] Arents J., Greitans M. (2022). Smart Industrial Robot Control Trends, Challenges and Opportunities within Manufacturing. Appl. Sci..

[15] Macdonald F.L.A., Lepora N.F., Conradt J., Ward-Cherrier B. (2022). Neuromorphic Tactile Edge Orientation Classification in an Unsupervised Spiking Neural Network. Sensors.

[16] Bing Z., Meschede C., Röhrbein F., Huang K., Knoll A.C. (2018). A Survey of Robotics Control Based on Learning-Inspired Spiking Neural Networks. Front. Neurorobot..

[17] Pietrzak P., Szczęsny S., Huderek D., Przyborowski Ł. (2023). Overview of Spiking Neural Network Learning Approaches and Their Computational Complexities. Sensors.

[18] Juárez-Lora A., García-Sebastián L.M., Ponce-Ponce V.H., Rubio-Espino E., Molina-Lozano H., Sossa H. (2022). Implementation of Kalman Filtering with Spiking Neural Networks. Sensors.

[19] Morris R.G.D.O. (1999). Hebb: The Organization of Behavior, Wiley: New York; 1949. Brain Res. Bull..

[20] Chen X., Zhu W., Dai Y., Ren Q. A Bio-Inspired Spiking Neural Network for Control of A 4-DoF Robotic Arm. Proceedings of the 2020 15th IEEE Conference on Industrial Electronics and Applications (ICIEA).

[21] Zaidel Y., Shalumov A., Volinski A., Supic L., Ezra Tsur E. (2021). Neuromorphic NEF-Based Inverse Kinematics and PID Control. Front. Neurorobot..

[22] Krakhmalev O., Krakhmalev N., Gataullin S., Makarenko I., Nikitin P., Serdechnyy D., Liang K., Korchagin S. (2021). Mathematics Model for 6-DOF Joints Manipulation Robots. Mathematics.

[23] Krakhmalev O., Korchagin S., Pleshakova E., Nikitin P., Tsibizova O., Sycheva I., Liang K., Serdechnyy D., Gataullin S., Krakhmalev N. (2021). Parallel Computational Algorithm for Object-Oriented Modeling of Manipulation Robots. Mathematics.

[24] Bellec G., Salaj D., Subramoney A., Legenstein R., Maass W. (2018). Long Short-Term Memory and Learning-to-Learn in Networks of Spiking Neurons. Proceedings of the Advances in Neural Information Processing Systems, Montreal, QC, Canada, 3–8 December 2018.

[25] Bellec G., Scherr F., Subramoney A., Hajek E., Salaj D., Legenstein R., Maass W. (2020). A Solution to the Learning Dilemma for Recurrent Networks of Spiking Neurons. Nat. Commun..

[26] Traub M., Legenstein R., Otte S. (2021). Many-Joint Robot Arm Control with Recurrent Spiking Neural Networks. arXiv.

[27] Lee J.H., Delbruck T., Pfeiffer M. (2016). Training Deep Spiking Neural Networks Using Backpropagation. Front. Neurosci..

[28] Massa R., Marchisio A., Martina M., Shafique M. An Efficient Spiking Neural Network for Recognizing Gestures with a DVS Camera on the Loihi Neuromorphic Processor. Proceedings of the 2020 International Joint Conference on Neural Networks (IJCNN).

[29] Hunsberger E., Eliasmith C. (2016). Training Spiking Deep Networks for Neuromorphic Hardwar. arXiv.

[30] Schuman C.D., Kulkarni S.R., Parsa M., Mitchell J.P., Date P., Kay B. (2022). Opportunities for Neuromorphic Computing Algorithms and Applications. Nat. Comput. Sci..

[31] Aaron Zeglen M. (2022). Amygdala Modeling with Context and Motivation Using Spiking Neural Networks for Robotics Applications. Master’s Thesis.

[32] Yamazaki K. (2020). Towards Sensorimotor Coupling of a Spiking Neural Network and Deep Reinforcement Learning for Robotics Application. Bachelor’s Thesis.

[33] Yamazaki K., Vo-Ho V.-K., Bulsara D., Le N. (2022). Spiking Neural Networks and Their Applications: A Review. Brain Sci..

[34] Wang S., Chen X., Huang X., Wei Zhang D., Zhou P. (2020). Neuromorphic Engineering for Hardware Computational Acceleration and Biomimetic Perception Motion Integration. Adv. Intell. Syst..

[35] Izhikevich E.M. (2004). Which Model to Use for Cortical Spiking Neurons?. IEEE Trans. Neural Netw..

[36] Shama F., Haghiri S., Imani M.A. (2020). FPGA Realization of Hodgkin-Huxley Neuronal Model. IEEE Trans. Neural Syst. Rehabil. Eng..

[37] Giannari A.G., Astolfi A. (2022). Model Design for Networks of Heterogeneous Hodgkin–Huxley Neurons. Neurocomputing.

[38] Dora S., Kasabov N. (2021). Spiking Neural Networks for Computational Intelligence: An Overview. Big Data Cogn. Comput..

[39] Lu S., Xu F. (2022). Linear Leaky-Integrate-and-Fire Neuron Model Based Spiking Neural Networks and Its Mapping Relationship to Deep Neural Networks. Front. Neurosci..

[40] Kim J., Choi Y.I., Sohn J., Kim S.-P., Jung S.J. (2023). Modeling Long-Term Spike Frequency Adaptation in SA-I Afferent Neurons Using an Izhikevich-Based Biological Neuron Model. Exp. Neurobiol..

[41] Xiao S., Liu W., Guo Y., Yu Z. (2020). Low-Cost Adaptive Exponential Integrate-and-Fire Neuron Using Stochastic Computing. IEEE Trans. Biomed. Circuits Syst..

[42] Carlu M., Chehab O., Dalla Porta L., Depannemaecker D., Héricé C., Jedynak M., Köksal Ersöz E., Muratore P., Souihel S., Capone C. (2020). A Mean-Field Approach to the Dynamics of Networks of Complex Neurons, from Nonlinear Integrate-and-Fire to Hodgkin–Huxley Models. J. Neurophysiol..

[43] Agebure M.A., Wumnaya P.A., Baagyere E.Y. (2021). A Survey of Supervised Learning Models for Spiking Neural Network. Asian J. Res. Comput. Sci..

[44] Hong C., Wei X., Wang J., Deng B., Yu H., Che Y. (2020). Training Spiking Neural Networks for Cognitive Tasks: A Versatile Framework Compatible with Various Temporal Codes. IEEE Trans. Neural Netw. Learn. Syst..

[45] Zenke F., Ganguli S. (2018). SuperSpike: Supervised Learning in Multilayer Spiking Neural Networks. Neural Comput..

[46] Fernández J.G., Hortal E., Mehrkanoon S. Towards Biologically Plausible Learning in Neural Networks. Proceedings of the 2021 IEEE Symposium Series on Computational Intelligence (SSCI).

[47] Shi C., Wang T., He J., Zhang J., Liu L., Wu N. (2021). DeepTempo: A Hardware-Friendly Direct Feedback Alignment Multi-Layer Tempotron Learning Rule for Deep Spiking Neural Networks. IEEE Trans. Circuits Syst. II Express Briefs.

[48] Wang S., Li C. A Supervised Learning Algorithm to Binary Classification Problem for Spiking Neural Networks. Proceedings of the 2021 8th International Conference on Information, Cybernetics, and Computational Social Systems (ICCSS).

[49] Hazan A., Ezra Tsur E. (2022). Neuromorphic Neural Engineering Framework-Inspired Online Continuous Learning with Analog Circuitry. Appl. Sci..

[50] DeWolf T., Stewart T.C., Slotine J.-J., Eliasmith C. (2016). A Spiking Neural Model of Adaptive Arm Control. Proc. R. Soc. B Biol. Sci..

[51] Joseph G.V., Pakrashi V. (2022). Spiking Neural Networks for Structural Health Monitoring. Sensors.

[52] DeWolf T. (2015). A Neural Model of the Motor Control System. Ph.D. Thesis.

[53] Stewart T., Eliasmith C. (2012). Compositionality and Biologically Plausible Models. The Oxford Handbook of Compositionality.

[54] Bekolay T., Bergstra J., Hunsberger E., Dewolf T., Stewart T., Rasmussen D., Choo X., Voelker A., Eliasmith C. (2014). Nengo: A Python Tool for Building Large-Scale Functional Brain Models. Front. Neuroinforma..

[55] Sharma S., Aubin S., Eliasmith C. (2016). Large-Scale Cognitive Model Design Using the Nengo Neural Simulator. Biol. Inspired Cogn. Archit..

[56] Kern J., Marrero D., Urrea C. (2023). Fuzzy Control Strategies Development for a 3-DoF Robotic Manipulator in Trajectory Tracking. Processes.

